# Chemotherapy Associated Bilateral Secondary Spontaneous Pneumothorax

**DOI:** 10.7759/cureus.28478

**Published:** 2022-08-27

**Authors:** Randeep Singh, Ruchi Dua, Nyrvan Baishya, Sharad P Dahal

**Affiliations:** 1 Pulmonary and Critical Care Medicine, All India Institute of Medical Sciences, Rishikesh, IND

**Keywords:** oncology, breast carcinoma, lung metastasis, chemotherapy-related toxicity, bilateral spontaneous pneumothorax

## Abstract

Chemotherapy-associated pneumothorax is a rarely encountered oncological emergency. Herein, we present a rare chemotherapy-associated bilateral secondary spontaneous pneumothorax case of a man in his 60s with invasive breast carcinoma after four cycles of chemotherapy. He presented to our emergency department with acute onset dyspnea and left-sided pleuritic chest pain. A chest X-ray showed a left-sided pneumothorax, and an intercostal chest tube (ICT) was inserted with underwater seal drainage. After three days, he complained of sudden onset right-sided chest pain and increased dyspnea. A repeat chest X-ray revealed right-sided pneumothorax, which was managed with ICT again. Bilateral pleurodesis was done after a repeat chest x-ray showed complete lung re-expansion. The patient was doing well with no recurrence of pneumothorax after three months of follow-up. Male breast cancer is uncommon, and presentation with bilateral secondary spontaneous pneumothorax is rare. This case is reported as a rare complication of chemotherapy-associated bilateral spontaneous pneumothorax.

## Introduction

Spontaneous pneumothorax is a severe but uncommon complication following chemotherapy for malignant diseases. It has been previously reported after chemotherapy or radiotherapy [1-3]. Malignancy-related pneumothorax accounts for about 0.05% of all pneumothorax cases [4], but it has diagnostic and therapeutic implications. Rapid regression of tumors following chemotherapy has been thought to be one of the possible causes of spontaneous pneumothorax post-chemotherapy [5,6]. Spontaneous pneumothoraces occur more often in metastatic osteogenic sarcomas, germ cell tumors, and lymphomas [7,8]. Male breast cancer is uncommon, and presentation with bilateral secondary spontaneous pneumothorax is rare.

## Case presentation

**Figure 1:**
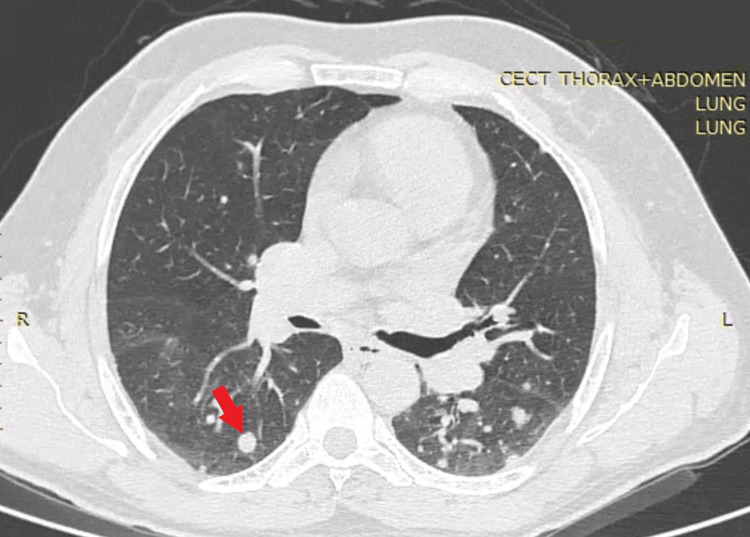
CT Chest before starting chemotherapy showed bilateral parenchymal metastatic nodules(red arrow)

**Figure 2:**
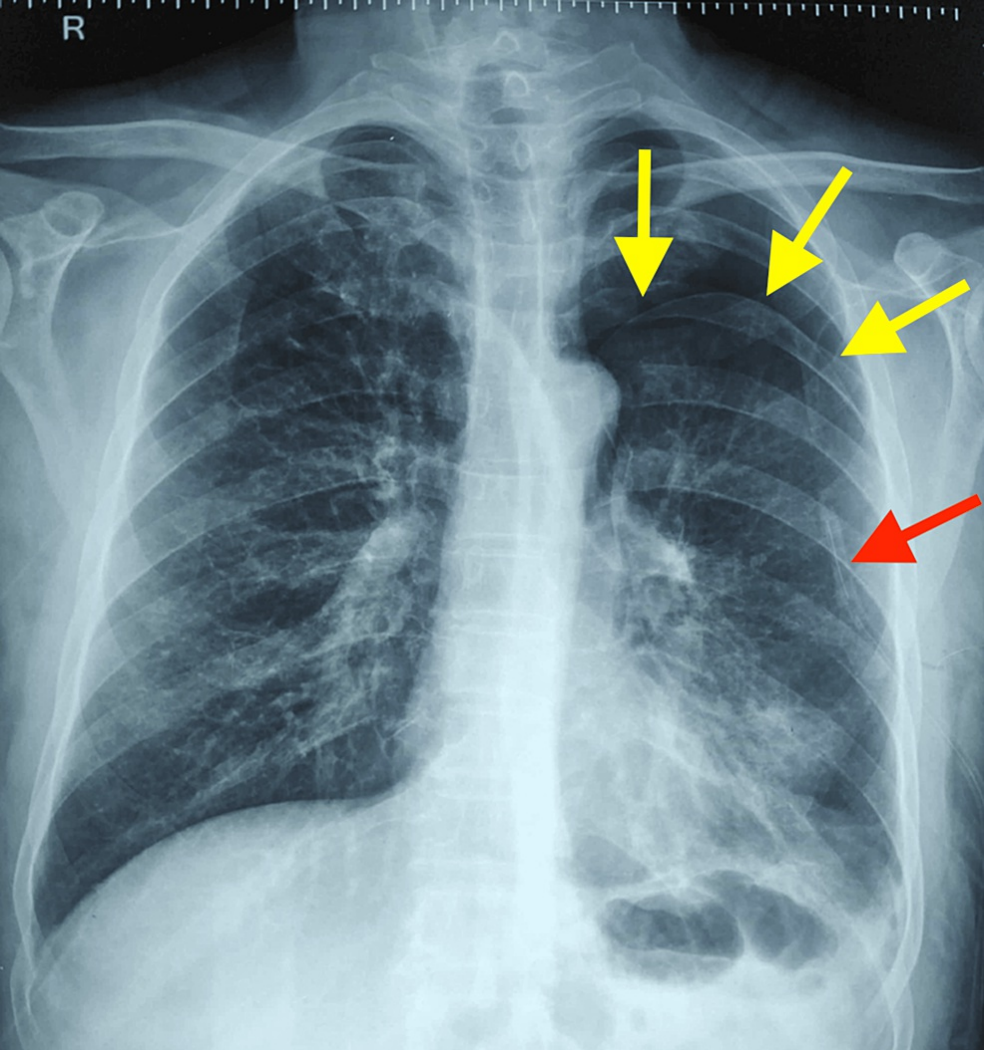
Chest X-ray showed left-sided pneumothorax (yellow arrows) and ICT in situ (red arrow).

**Figure 3:**
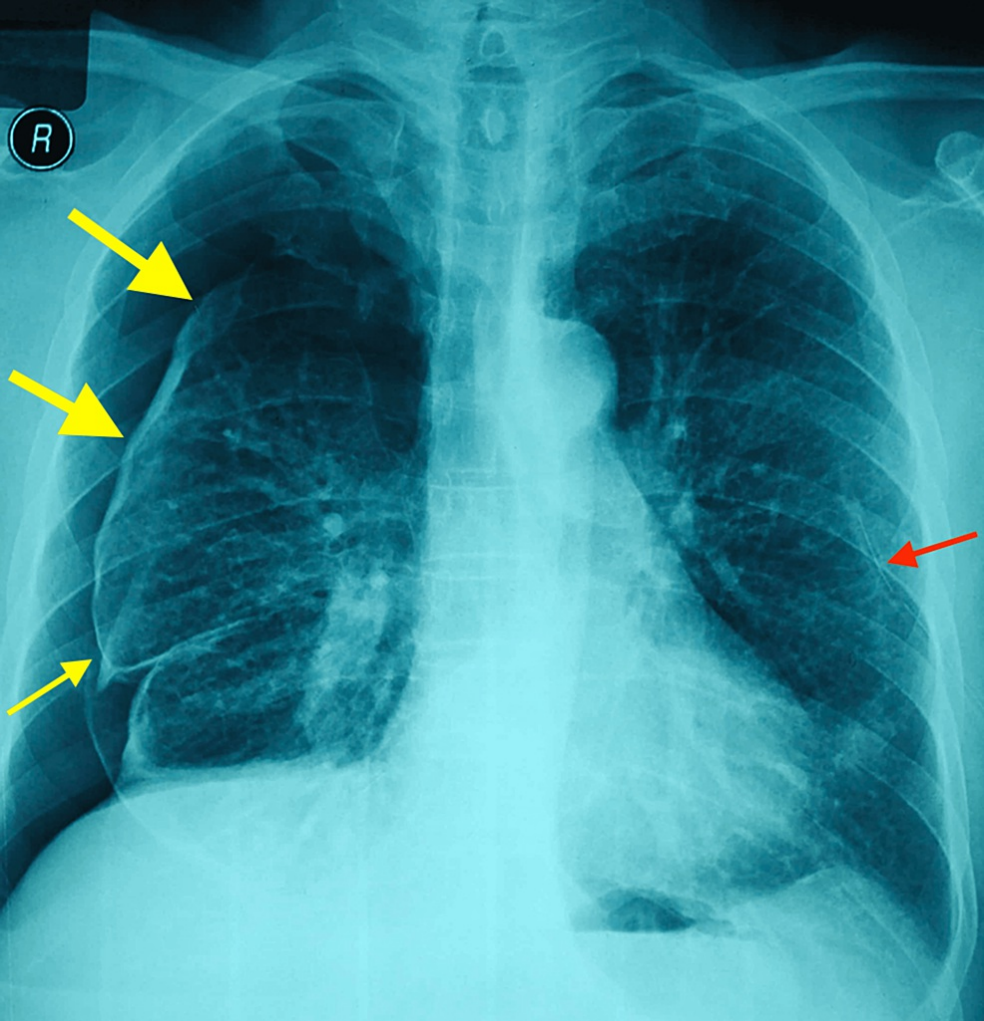
Day 3 Chest X-ray, showed right-sided pneumothorax (yellow arrows) with left-sided ICT in situ (red arrow).

A man in his 60s, an ex-smoker with 15 pack-year smoking history with invasive carcinoma of the left breast, presented to the emergency department with complaints of left-sided pleuritic chest pain for the last day and sudden onset of breathlessness, grade-4 according to modified medical research council (mMRC) scale. There was no history of fever, hemoptysis, diaphoresis, or skin rashes. The patient was diagnosed with invasive carcinoma left breast (cT2N1M1) 5 months back. The cancer was classified as Nottingham Grade-3 with ER (estrogen receptor) positive (Allred score 5-3) and PR (progesterone receptor) positive (Allred score 4-2) status. Contrast-enhanced computed tomography (CECT) of the thorax done five months back showed bilateral multiple pulmonary nodules suggestive of pulmonary metastasis, bilateral subpleural nodules, and minimal emphysema-like changes in the lung apices (Figure 1, Video 1). The patient had no respiratory complaints at that time. The patient received four cycles of Doxorubicin 95mg and Cyclophosphamide 950mg and was on tamoxifen 20mg daily. The patient did not receive any radiotherapy. On clinical examination, the patient was tachypneic with a respiratory rate of 28 breaths/min, using accessory muscles of respiration and oxygen saturation of 80% on room air. On chest examination, there was a hyper-resonant note on percussion with decreased intensity of breath sounds over the left thorax. Chest X-ray showed left-sided pneumothorax. A left-sided ICT was inserted in the emergency; post ICT insertion, a chest X-ray showed partial lung re-expansion (Figure 2), along with clinical improvement. After three days, he again complained of right-sided diffuse chest pain with increased breathlessness. A repeat chest x-ray showed pneumothorax on the right side (Figure 3).

**Video 1:** CT Chest video before starting Chemotherapy Showing bilateral parenchymal metastatic nodules.

Treatment

**Figure 4:**
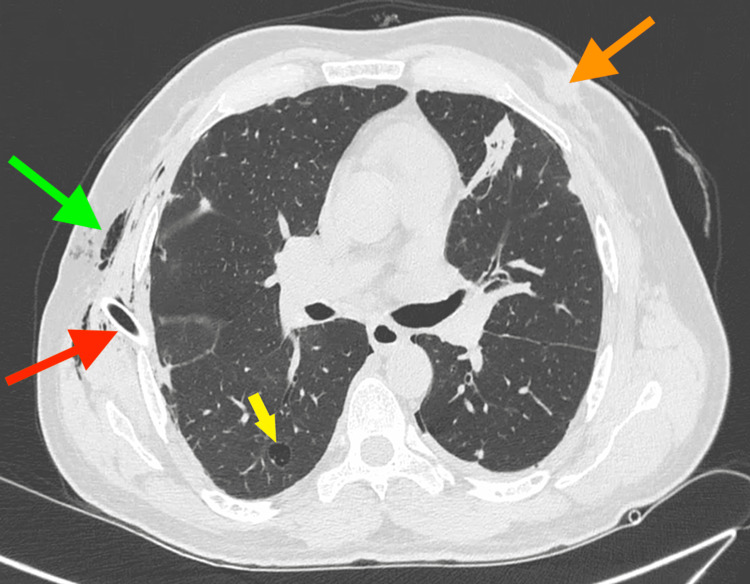
CT Chest image after receiving chemotherapy showed cystic change in the parenchymal nodule (yellow arrow), with right-sided surgical emphysema (green arrow) and right-sided ICT in situ (red arrow). A lesion can also be seen in the left breast (orange arrow).

**Figure 5:**
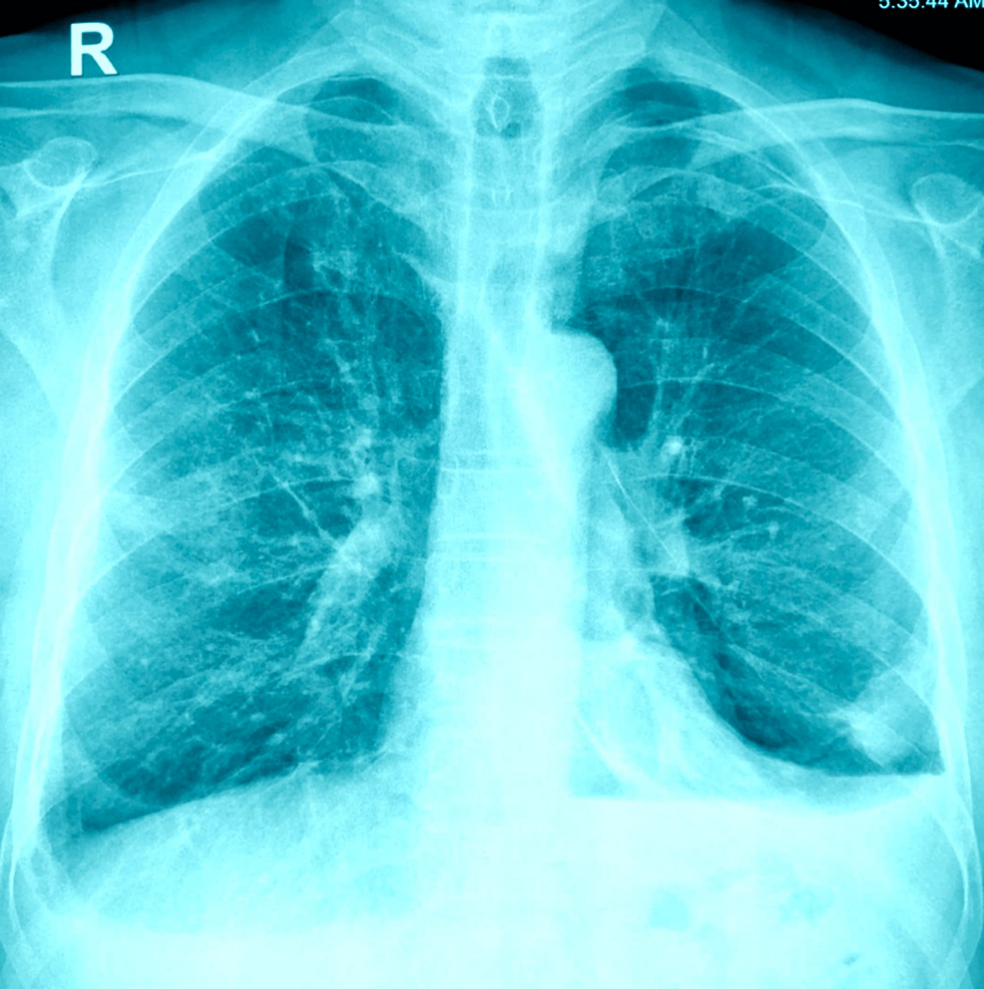
Post-Pleurodesis Chest X-ray.

The patient was managed with right-sided ICT insertion. A repeat CECT thorax was done, which showed pulmonary nodules, few nodules with cavitation, and ICT in situ (Figure 4, Video 2). Complete re-expansion of both lungs was achieved by day six, and bilateral iodopovidone pleurodesis was done as the patient was not affording talc. Post-pleurodesis chest x-rays showed no evidence of any residual pneumothorax or significant pleural effusion (Figure 5). Tamoxifen 20mg was continued on discharge as advised by the oncology team.

**Video 2:** CT Chest video after receiving Chemotherapy.

Outcome and follow-up

The patient was doing well at the three-month follow-up and had no recurrence of pneumothorax.

## Discussion

Primary or metastatic lung malignancies are an uncommon cause of secondary spontaneous pneumothorax (SSP). Commonly reported malignancies causing spontaneous pneumothorax include primary lung cancers [6], sarcomas [9], and germ cell tumors [10]. Uncommonly endometrial cancer [11], mediastinal lymphoma [12], renal cell carcinoma [13], and angiosarcoma [14] have also been reported to cause SSP. Breast cancers with SSP have rarely been reported [15] in women. Male breast cancer is uncommon, and presenting with bilateral SSP is even more so.

The mechanism complicating pneumothorax in patients with primary and metastatic lung malignancies is poorly understood. Several hypotheses have been proposed for the underlying pathophysiology of pneumothoraces in cancer patients. SSP may result from underlying pathological lung abnormalities due to either tumor progression or emphysematous changes secondary to common etiologies like smoking. Another postulated hypothesis proposes that rapid regression of tumor or necrosis related to chemotherapy or targeted therapy may cause SSP [16,17]. Bronchial wall compression by tumor resulting in air trapping and subsequent rupture, pleural and vascular invasion by the tumor leading to bronchopleural fistula, and increased intra-thoracic pressure due to chemotherapy-related emesis is the postulated theories [18]. In our case, the previous CT thorax done five months back showed multiple bilateral contrast-enhanced lung nodules suggestive of pulmonary metastasis. The latest CT scan (Figure 5, Video 2) done during the admission showed a cystic lesion likely due to regression of previous metastatic pulmonary nodule following chemotherapy. 

The usual time frame, as reported in other case reports, is consistent with this case, wherein it occurred after four cycles of chemotherapy. Many chemotherapeutic agents (actinomycin-D, bevacizumab, bleomycin, carboplatin, cisplatin, cyclophosphamide, docetaxel, doxorubicin, erlotinib, etoposide, gefitinib, gemcitabine, paclitaxel, pemetrexed, oxaliplatin, vinblastine, vinorelbine) [19,20] have been implicated in causing pneumothorax. In our case, both doxorubicin and cyclophosphamide were used, and both drugs were incriminated as potential causative agents for pneumothorax. Further studies are needed to elucidate the mechanisms underlying the etiology of chemotherapy-associated pneumothorax and whether this toxicity is dose-related. Optimal treatment, whether surgical or ICT and chemical pleurodesis, is also not precisely known, with isolated case reports utilizing both management strategies. The occurrence of spontaneous pneumothorax is prone to recurrence and carries a poorer prognosis, especially with sarcomas [20].

## Conclusions

This case report highlights that chemotherapy-associated pneumothorax should be included among the differentials of oncology patients presenting with acute onset dyspnea. Regression of metastatic nodules along with cystic degeneration due to chemotherapy may be one of its possible etiologies. ICT with chemical pleurodesis is an effective strategy for managing and preventing pneumothorax recurrence. In conclusion, chemotherapy-associated spontaneous pneumothorax is a rare but potentially life-threatening complication requiring prompt intervention. Such cases usually have poor prognoses and are prone to recurrence, necessitating the need for pleurodesis.
